# Structural Analysis of the Essential Resuscitation Promoting Factor YeaZ Suggests a Mechanism of Nucleotide Regulation through Dimer Reorganization

**DOI:** 10.1371/journal.pone.0023245

**Published:** 2011-08-17

**Authors:** Inci Aydin, Yumiko Saijo-Hamano, Keiichi Namba, Connor Thomas, Anna Roujeinikova

**Affiliations:** 1 Department of Microbiology and Department of Biochemistry and Molecular Biology, Monash University, Clayton, Victoria, Australia; 2 Graduate School of Frontier Biosciences, Osaka University, Suita, Osaka, Japan; 3 School of Molecular and Biomedical Science, University of Adelaide, Adelaide, South Australia, Australia; University of Cambridge, United Kingdom

## Abstract

**Background:**

The *yeaZ* gene product forms part of the conserved network YjeE/YeaZ/YgjD essential for the survival of many Gram-negative eubacteria. Among other as yet unidentified roles, YeaZ functions as a resuscitation promoting factor required for survival and resuscitation of cells in a viable but non-culturable (VBNC) state.

**Methodology/Principal Findings:**

In order to investigate in detail the structure/function relationship of this family of proteins we have performed X-ray crystallographic studies of *Vibrio parahaemolyticus* YeaZ. The YeaZ structure showed that it has a classic actin-like nucleotide-binding fold. Comparisons of this crystal structure to that of available homologues from *E. coli*, *T. maritima* and *S. typhimurium* revealed two distinctly different modes of dimer formation. In one form, prevalent in the absence of nucleotide, the putative nucleotide-binding site is incomplete, lacking a binding pocket for a nucleotide base. In the second form, residues from the second subunit complete the nucleotide-binding site. This suggests that the two dimer architectures observed in the crystal structures correspond to a free and a nucleotide-bound form of YeaZ. A multiple sequence alignment of YeaZ proteins from different bacteria allowed us to identify a large conserved hydrophobic patch on the protein surface that becomes exposed upon nucleotide-driven dimer re-arrangement. We hypothesize that the transition between two dimer architectures represents the transition between the ‘on’ and ‘off’ states of YeaZ. The effect of this transition is to alternately expose and bury a docking site for the partner protein YgjD.

**Conclusions/Significance:**

This paper provides the first structural insight into the putative mechanism of nucleotide regulation of YeaZ through dimer reorganization. Our analysis suggests that nucleotide binding to YeaZ may act as a regulator or switch that changes YeaZ shape, allowing it to switch partners between YjeE and YgjD.

## Introduction

The *yeaZ* gene product forms part of the conserved network YjeE/YeaZ/YgjD essential for survival of many eubacteria [1], [2]. Studies in *Salmonella* [3] and *V. parahaemolyticus* (C. Thomas, personal communication, 19 November 2010) demonstrated that among other as yet unidentified roles, YeaZ functions as a resuscitation promoting factor required for cells to be able to survive in, and exit from, a VBNC state. Many pathogenic bacteria enter the VBNC state as a response to stress (e.g. starvation of nutrients, change in osmotic and oxygen concentrations or temperature) [4]. The marine enteropathogen *V. parahaemolyticus*, for example, enters a VBNC state at temperatures below 15°C, that correspond with typical winter seawater temperatures as well as temperatures used for storage of seafood, as a survival strategy [5]. VBNC cells exhibit antibiotic resistance and retain the ability to attach and persist in their environment. Exposing *V. parahaemolyticus* VBNC cells to a temperature upshift leads to resuscitation; the cells regain culturability and renewed ability to cause infection. The requirement of YeaZ for persistence within, and exit from, the VBNC state suggests that it might be a new promising target for antimicrobial agents.

Structural studies on YeaZ homologues from *E. coli* (*Ec*YeaZ [6]), *Thermotoga maritima* (*Tm*YeaZ [7]) and *S. typhimurium* (*St*YeaZ [8]) have revealed that these proteins adopt a two-lobed HSP70/actin-like fold. Their structure is distinctly different from that of resuscitation promoting factors found in Gram-positive bacteria [8].

*Ec*YeaZ has been shown to interact with the conserved essential proteins YjeE and YgjD, with YgjD being the preferred interaction partner [2]. YeaZ acted as a protease that specifically degrades YgjD in *in vitro* experiments [2] and it has been suggested that YeaZ can post-translationally regulate cellular pools of YgjD *via* proteolytic degradation. Complementation of the *E. coli ygjD* essentiality phenotype with orthologs from *Bacillus subtilis* required coexpression of the *B. subtilis ygjD/yeaZ* pair, indicating that the YgjD function is dependent on YeaZ [9]. Although the biochemical pathways dependent on YeaZ/YgjD/YjeE have yet to be established, the requirement of YgjD for t6A biosynthesis in *E. coli* suggests a functional link between this network and the tRNA synthesis control system [9], [10]. Purified *E. coli* YeaZ, YjeE and YgjD are dimers in solution [2]. Although there is no detailed experimental information about the mode of association between YeaZ and YgjD or YeaZ and YjeE, the nucleotide-binding fold of YeaZ suggests that its interactions and activity are likely to be regulated by a nucleotide.

In this paper, we report the crystal structure of *V. parahaemolyticus* YeaZ (*Vp*YeaZ) to 3.1 Å resolution. Comparisons of this crystal structure to that of YeaZ from *E. coli*, *T. maritima* and *S. typhimurium* reveals two distinctly different modes of dimerization, only one of which has a complete putative nucleotide-binding site. Transitions between the two forms expose a large conserved hydrophobic region on the protein surface, suggesting a model in which nucleotide binding to YeaZ is dependent on and regulated by binding to its partner protein YgjD.

## Results

### The Overall Fold of YeaZ

**Figure 1:**
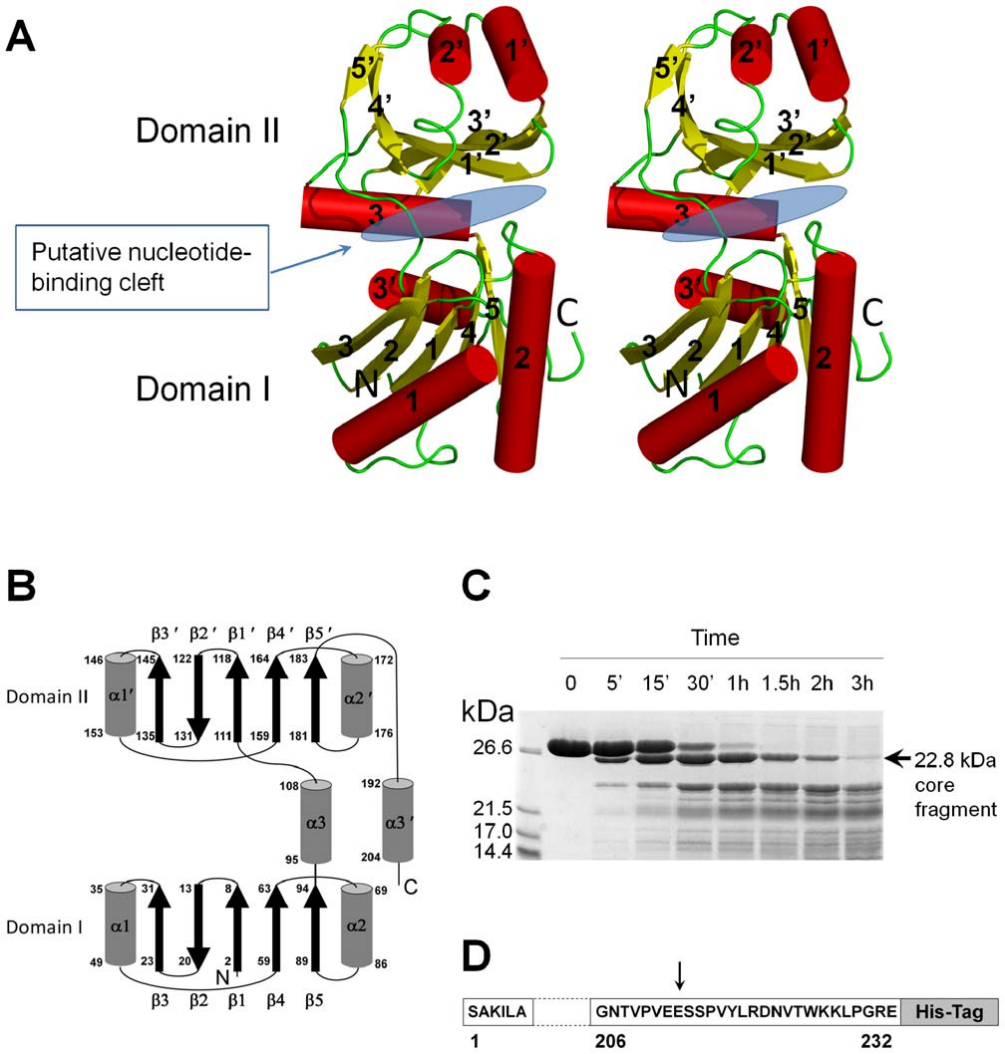
A: Stereo diagram of the structure of the *Vp*YeaZ monomer. Each element of the secondary structure is labeled. Domains I and II and the putative nucleotide-binding cleft are identified. The figure was prepared using PyMOL [11]. B: The topology of the secondary structure elements. Residue numbers are indicated at the start and end of each secondary structure element. C: SDS-PAGE showing the time-course of VpYeaZ digestion by Glu-C protease. The positions of molecular mass markers are shown to the left. The arrow indicates a relatively stable C-terminally truncated fragment. D: Amino acid sequence of VpYeaZ with Glu-C-sensitive site identified in this study shown by the arrow.

The structure of *Vp*YeaZ has been solved to 3.1 Å resolution revealing a two domain architecture with a duplicated βββαβαβα secondary structure (Figs. 1A,B). The domains I and II have a common fold that comprises a five-stranded mixed β-sheet (order 32145; strand 2 is antiparallel to the rest) surrounded by three α-helices. The folding pattern of the secondary structure elements is identical to that of the core of actin-like nucleotide-binding proteins (the superfamily referred to as ASKHA (acetate and sugar kinases/Hsc70/actin) [12] or HALF (Hsp70/actin-like fold) in the literature [13], [14]. This suggests that, similar to other members of this family, the cleft between domains I and II is likely to accommodate a nucleotide-binding site.

The asymmetric unit contains four subunits, each comprising residues 2–213. The N-terminal residue S1 is disordered. The 19 C-terminal residues are missing, most likely having been proteolytically removed during crystallization (see Materials and Methods). To investigate the boundaries of a stable core resistant to proteolysis, we carried out digests of *Vp*YeaZ with Glu-C protease which cleaves at the C-terminal side of Glu residues. The time-course of degradation, as monitored by SDS-PAGE (Fig. 1C) and Western blotting analysis (data not shown), demonstrated accumulation of a relatively stable truncated variant lacking C-terminal His-tag before further degradation into shorter products. To determine the proteolytic cleavage sites the truncated variant was isolated by reverse-phase chromatography and its molecular mass was measured by matrix-assisted laser desorption ionization-time-of-flight mass spectrometry, yielding the value of 22,817±100 Da. N-terminal sequencing confirmed that the N-terminus in the truncated variant was intact. The truncated variant was identified as fragment 1–213 (Fig. 1D) with excellent agreement between the experimental and the calculated (22,810 Da) mass values. This analysis suggested that approximately 20 C-terminal residues of *Vp*YeaZ are flexible and accessible to protease and that the remainder has a compact and stable fold. This conclusion is consistent with the observation that the crystals were formed by fragment 1–213 rather than full-length protein 1–232.

### Structural comparisons to other members of the ASKHA family and characterization of the putative nucleotide-binding site

**Figure 2:**
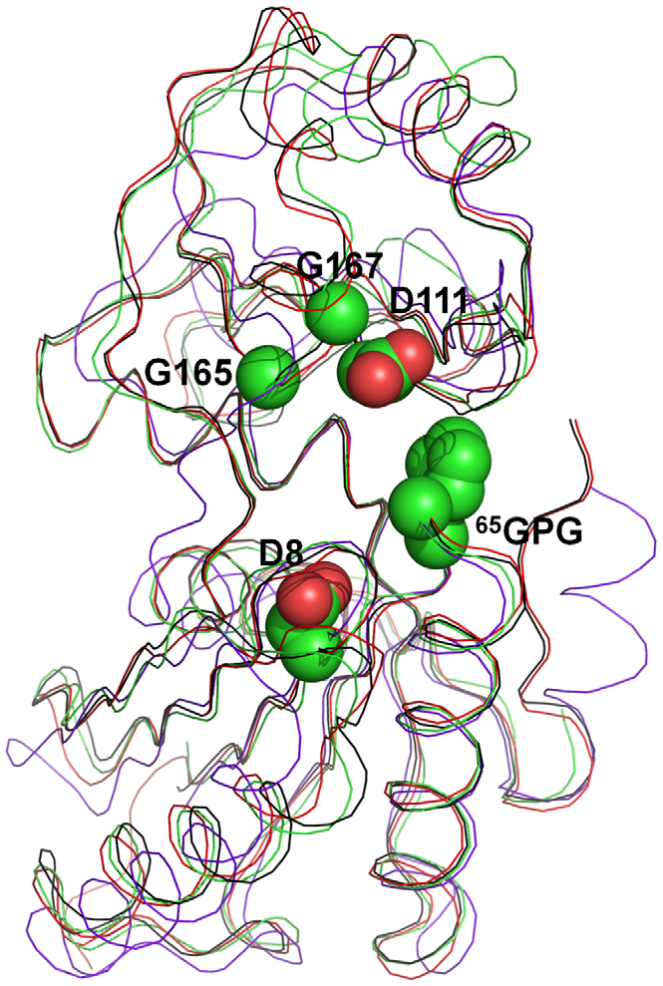
The structural overlap between *Vp*YeaZ (green), *Tm*YeaZ (purple), *Ec*YeaZ (red) and *St*YeaZ (black). The side chains of the conserved and semi-conserved residues likely to be implicated in nucleotide binding are shown for *Vp*YeaZ.

Comparison of the crystal structure of the *Vp*YeaZ monomer with that of homologous proteins from *E. coli* (PDB code 1okj [6]), *T. maritima* (PDB code 2a6a [7]) and *S. typhimurium* (PDB code 2gel [8]) showed that the four structures are closely similar, including the relative orientation of the two domains (Fig. 2). The structures of *Ec*YeaZ and *St*YeaZ can be superimposed with that of *Vp*YeaZ over the 198 of 212 C_α_ atoms with an rmsd of 0.9 Å, with the three sequences showing 54% sequence identity over the aligned amino acid residues. Good overlap of the structures is not surprising, considering the high sequence identity. The structures of *Vp*YeaZ and *Tm*YeaZ can be superimposed over 133 C_α_ atoms with an rmsd of 1.3 Å showing 24% sequence identity over the aligned residues.

The distinct biological functions of functionally diverse proteins from the ASKHA superfamily are thought to be mediated by subdomains inserted at characteristic topological positions between β3 and α1, β4 and α2 and/or α2 and β5 of domain I, and β3′ and α1′ of domain II [13]. These inserts play a role in oligomerization, substrate and effector binding [12], [13]. Remarkably, YeaZ appears to be unique amongst other members of ASKHA superfamily in that it lacks these inserts; the lengths of loops β3α1, β4α2, α2β5 and β3′α1′ in *Vp*YeaZ do not exceed five residues (Fig. 1B). Thus, the YeaZ structure represents the minimal functional core of the ASKHA superfamily.

**Figure 3:**
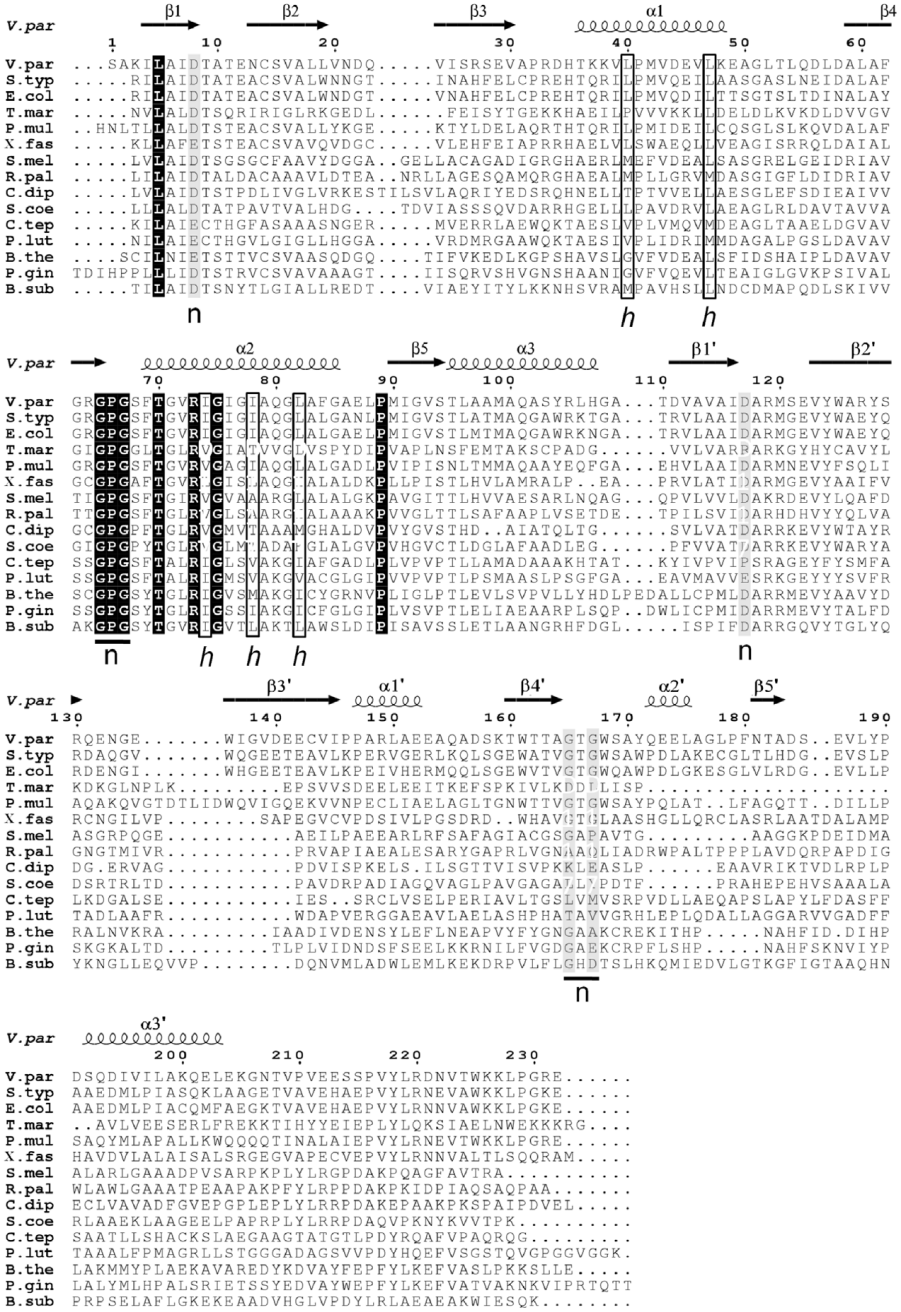
A sequence alignment of YeaZ homologues in *V. parahaemolyticus* (V. par), *S. typhimurium* (S. typ), *E. coli* (E. col), *T. maritima* (T. mar), *Pasteurella multocida* (P. mul), *Xylella fastidiosa* (X. fas), *Sinorhizobium meliloti* (S. mel), *Rhodopseudomonas palustris* (R. pal), *Corynebacterium diphtheria* (C. dip), *Streptomyces coelicolor* (S. coe), *Chlorobium tepidum* (C. tep), *Pelodictyon luteolum* (P. lut), *Bacteroides thetaiotaomicron* (B. the), *Porphyromonas gingivalis* (P. gin) and *Bacillus subtilis* (B. sub). The elements of the secondary structure and the sequence numbering for *Vp*YeaZ are shown above the alignment. The fully conserved residues are shown by a black box with reverse type. The position of the conserved and semi-conserved residues which can be identified in the nucleotide-binding site of proteins belonging to the ASKHA family, are highlighted by black and gray boxes respectively, and marked with “n” underneath the aligned sequences. The residues forming a hydrophobic surface patch which becomes exposed upon dimer re-organisation are enclosed by a box and marked with “*h*” underneath the alignment. Alignment was carried out using the ClustalW server [16]. The figure was created using ESPript [17].

Despite similar core fold, the sequences of functionally diverse proteins from the ASKHA superfamily show very little conservation mainly characterized by the presence of one or more glycine-rich or glycine-containing loops associated with nucleotide binding [15]. The turn between β1 and β2 is thought to play a critical role in nucleotide binding, with main chain nitrogens of the turn interacting with the phosphates [13]. Structure and sequence comparisons showed that in YeaZ, the glycine in the conserved motif DXG in loop β1β2 is replaced by a small side-chain residue, such as alanine, valine or serine (A10 in *Vp*YeaZ), whilst the aspartate (D8 in *Vp*YeaZ) is strongly conserved (Fig. 3). The loop between β4′ and α2′ harbors a strongly conserved ^165^GXG motif (Figure 2B) that is believed to be involved in binding of the nucleotide base moiety [15]. YeaZ and a structurally similar yeast kinase-associated endopeptidase 1 (Kae1) share a strongly conserved aspartate D117 (*Vp*YeaZ numbering) that is involved in binding of the ribose moiety of the nucleotide in Kae1 [18], [19]. Furthermore, analysis of sequence conservation in YeaZ proteins from different bacteria identified an absolutely conserved glycine-rich motif ^65^GPGXXTGXR which is located in the cleft between domains I and II and is therefore likely to be important for recognition of the nucleotide phosphate moiety (Fig. 3). The position of the aforementioned residues in the YeaZ structure is shown in Fig. 2.

### Two possible modes of YeaZ dimerization and implications for nucleotide binding

**Figure 4:**
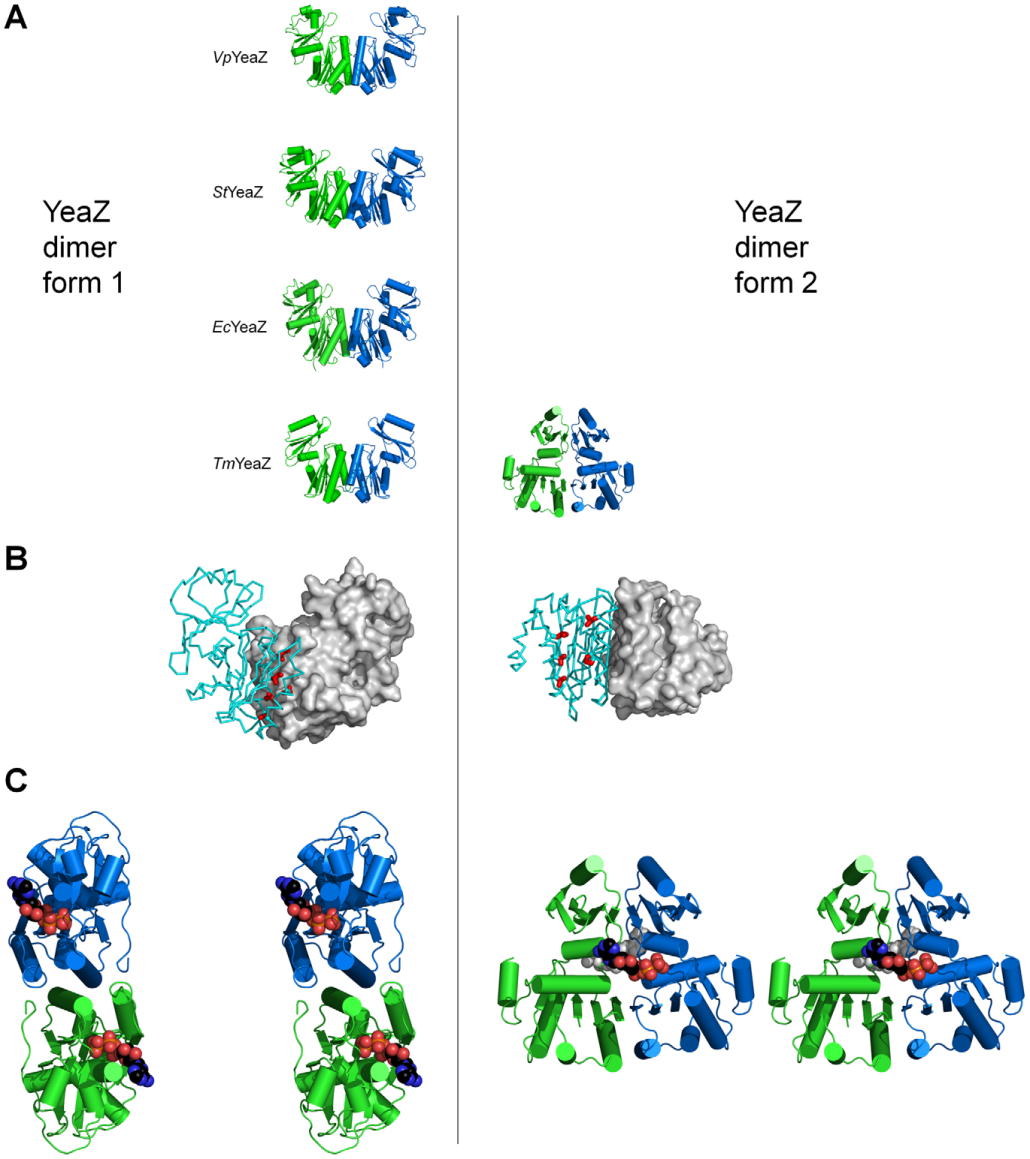
A: Comparison of form 1 and form 2 dimers observed in the crystal structures of *Vp*YeaZ, *St*YeaZ, *Ec*YeaZ and *Tm*YeaZ. B: Location of residues L40, L47, I74, I78, L82 (shown in red) at the interface of *Vp*YeaZ dimer (left) and positions of the equivalent residues in *Tm*YeaZ form 2 dimer (right). The residue positions are shown for one half of the dimer, with the other half represented by its molecular surface. C: A stereo diagram showing modeled positions of a nucleotide molecule in the interdomain cleft of the form 1 (left) and the form 2 dimers. The nucleotide (ATP) molecule has been positioned based on structural similarity between domains I in YeaZ and Kae1. The modelled nucleotide is shown in ball representation. In the model of the form 2 dimer complex one of the ATP molecules is shown in light grey for clarity of illustration.

We have earlier reported that *Vp*YeaZ is mostly dimeric in solution [20], in line with the previous gel-filtration studies on *E. coli* YeaZ in solution [2]. Analysis of the packing of *Vp*YeaZ monomers in the crystal lattice identified an obvious dimer with 2-fold symmetry and approximate dimensions of 30×45×80 Å (Fig. 4A). The subunits contact each other *via* domain I, which is more conserved than domain II according to analysis of sequence alignment (Fig. 3). The dimer interface is formed by residues from helices α1 and α2 from both subunits, which form a 4-helical bundle. Ten percent (1037 Å^2^) of the subunit accessible surface area is buried upon dimerization, which falls within the range found for other dimeric proteins [21]. We therefore conclude that *Vp*YeaZ is a dimer, both in solution and in crystalline environment.

Analysis of the molecule packing in the crystals of *Ec*YeaZ (PDB code 1okj [6]), *St*YeaZ (PDB code 2gel [8]) and *Tm*YeaZ (PDB code 2a6a [7]) suggested two possible modes of dimerization. One form (hereafter referred to as form 1), found in crystals of all homologues of known structure, is very similar to that seen in the *Vp*YeaZ crystals (Fig. 4A). The dimer interface in this form shields 9–10% of the monomer surface area and buries a region of strongly conserved hydrophobic residues L40, L47, I74, I78, L82 (*Vp*YeaZ numbering) (Fig. 4B). Sequence conservation at the interface and preservation of the dimer architecture in different homologues strongly suggested that this form of a dimer is physiologically relevant. The second possible mode of dimerization (in addition to the first one), where the β3 strands from the two domains I pack together in an anti-parallel manner to form an extended β-sheet, has been observed in the crystals of *Tm*YeaZ (form 2, Fig. 4A) [7]. In this mode, the dimer interface involves both domains I and II and buries a larger (1037 Å^2^, or 13%) surface per monomer than in the first form, prompting the suggestion that this form of the dimer may be more stable [7]. The hydrophobic surface region in the form 2 *Tm*YeaZ dimer, which is structurally equivalent to the (L47, I74, I78, L82) region in *Vp*YeaZ, is exposed to the solvent (Fig. 4B).

To understand the functional implications of the existence of two possible dimer architectures, the putative position of the bound nucleotide in YeaZ has been analysed by superimposing the coordinates of the more conserved domain I in *Vp*YeaZ and the crystal structure of the complex of structurally similar protein Kae1 with ATP (Fig. 4C) (PDB code 2ivo [19]). It is evident from this analysis that due to lack of insertions characteristic for other members of the ASKHA superfamily, the putative nucleotide-binding site in the YeaZ monomer and form 1 dimer is incomplete and lacks a binding pocket for a nucleotide base. It is known that rotation of domains I and II by about 30° accompanies nucleotide binding in some actin-like proteins [14], [15], [22]. However, it is not conceivable that a domain movement in YeaZ might create a well-defined binding pocket for the nucleotide moiety. In contrast, in the model for the nucleotide–bound dimer form 2, produced by superimposing domains I of the *Tm*YeaZ dimer and the Kae1 complex with ATP, residues from the second subunit complete the nucleotide-binding site (Fig. 4C). This suggests that the dimer architectures 1 and 2 observed in the crystal structures may correspond to a free and a nucleotide-bound form of YeaZ. The fact that, in the absence of a nucleotide, form 1 is prevalent in the crystals of YeaZ from different bacteria, supports this view.

## Discussion

The structure of *Vp*YeaZ confirmed that it is a member of the ASKHA superfamily of actin-like nucleotide-binding proteins characterized by two domains (I and II) that hold a nucleotide-binding site in the interdomain cleft. One significant difference between YeaZ and other AKSHA-fold proteins is the absence of additional subdomains that are normally inserted at the characteristic topological positions in both domain I and II. These subdomains are unique to the specific function of the proteins, contributing towards substrate and effector recognition and oligomerization. We therefore hypothesize that YeaZ requires one or more partner proteins (*e.g.* YjeE, YgjD) to be functional. This requirement may explain why interactions of *St*YeaZ on its own with nucleotide cofactor/substrate candidates could not be detected in ITC experiments [8].

Although the nature of a nucleotide recognised by YeaZ is not yet known, it is likely that signalling nucleotide-like molecules play a significant role in regulation of key proteins essential for the transition from the VBNC state to growth. A signalling molecule guanosine 5′-diphosphate 3′-diphosphate (ppGpp) in particular has been implicated in the resuscitation of the VBNC state [23]. In this respect, we should note that the AKSHA superfamily contains a pppGpp phosphohydrolase (GPPA) that regulates the conversion of pppGpp to ppGpp [24]. However, there is little similarity between GPPA and YeaZ beyond the conserved actin-like core.

In some bacterial species including *Mycobacterium leprae*, *Achromobacter xylosoxidans* and *Bordetella petrii*, the *yeaZ* gene is fused with the gene encoding ribosomal-protein-alanine N-acetyltransferase RimI. Acetyl-CoA and CoA are the substrate and the product of the RimI-catalysed reaction, respectively. Although at present there is no data in support of the possible functional link between YeaZ and RimI, it is interesting to speculate that the nucleotide binding site of YeaZ may accommodate the adenine nucleotide moiety of CoA or acetyl-CoA.

Our structural analysis identified two distinctly different modes of YeaZ dimer formation. In one form, prevalent in the absence of nucleotide, the putative nucleotide-binding site is incomplete and lacks a binding pocket for a nucleotide base. In the second form, an interface between the two subunits apparently completes a nucleotide-binding site. This analysis suggests that the two dimer architectures observed in the crystal structures correspond to a free and a nucleotide-bound form of YeaZ. In this model, nucleotide binding to YeaZ is dependent on and regulated by trans-acting elements from neighbouring subunits.

Our analysis suggests that nucleotide binding to YeaZ acts as a regulator or a switch that changes the shape of YeaZ and allows it to interact with different partner proteins. We have identified a large conserved hydrophobic region on the protein surface (L40, L47, I74, I78, L82) that is shielded at the interface of the form 1 dimer, but becomes exposed upon nucleotide-driven dimer re-arrangement. We hypothesize that the transition between two dimer architectures represents the transition between the ‘on’ and ‘off’ states of YeaZ. The effect of this transition is to alternately expose and bury a hydrophobic docking site for a partner protein YgjD, although we cannot exclude the possibility that a YeaZ/YgjD complex forms first, thereby allowing subsequent nucleotide binding to YeaZ. Furthermore, in the cellular pathway that involves alternative complex formation between YeaZ and either YjeE or YgjD (Handford *et al.*, 2009) nucleotide binding to YeaZ may act as a regulator that allows YeaZ to switch partners between YjeE and YgjD. The presented structural analysis provides a useful foundation for more systematic mutagenesis and biochemical studies with the aim to address this hypothesis.

## Materials and Methods

### Crystallization, Data Collection and Structure Determination

Purification of YeaZ from *V. parahaemolyticus* strain NCTC 10884, crystallization, data collection and phasing by molecular replacement have been previously described [20].

### Limited Proteolysis of YeaZ

Proteolysis of YeaZ was performed in 40 mM Tris/HCl buffer pH 8.0 at 298 K. In a typical reaction Glu-C (V8 protease; Roche Diagnostics) was added to a YeaZ solution at 1 mg/ml at a ratio of protease to YeaZ of 1∶300 (w/w). At various times 15 µl aliquots were removed for electrophoretic and Western blotting analysis. The reaction was stopped by adding SDS-PAGE loading dye, incubating the sample at 368 K for 3 min and freezing it.

### Isolation of Proteolytic Fragments

0.4 mg of YeaZ was incubated with Glu-C as above for 1 h (reaction volume 400 µl). The reaction was stopped by adding 400 µl of buffer A (0.065% trifluoroacetic acid (TFA), 2% acetonitrile in water). The stable core fragment was isolated from the mixture by reverse-phase chromatography on Resource RPC 3 ml column (GE Healthcare). Elution was achieved with a linear gradient from buffer A to buffer B (0.05% TFA in 80% acetonitrile) over 45 ml. Protein fragments were monitored in the effluent at 280 nm and confirmed by SDS-PAGE analysis.

### Matrix-assisted laser desorption ionization-time-of-flight mass spectrometry

1 µl of the purified stable core fragment was mixed with 1.0 µl of 50% (v/v) acetonitrile, 0.3% (v/v) trifluoroacetic acid, 10 mg/ml sinapinic acid, placed on the sample plate and allowed to dry. Molecular mass was analyzed by a mass spectrometer (Voyager DE/PRO, PerSeptive Biosystems) in linear mode.

### N-terminal sequencing of the stable core fragment

Proteins in SDS/polyacrylamide gel were transferred onto a polyvinylidene difluoride (PVDF) membrane (Bio-Rad) with a Hoefer transblotting apparatus and stained for 5 min with 0.025% (w/v) Coomassie brilliant blue in 40% (v/v) methanol. After destaining the PVDF membranes for 10 min with 50% (v/v) methanol, the band of interest was cut out and subjected to N-terminal amino acid sequence analysis (Center for Medical Research and Education, Osaka university).

### X-ray crystallographic refinement and analysis

Molecular replacement with PHASER [25] revealed that the asymmetric unit contains two YeaZ dimers that are related by a non-crystallographic two-fold axis, thus forming a p222 tetramer. The Fourier electron density maps inspected with the program COOT [26] showed no density for the C-terminal residues beyound E213 in all subunits in the asymmetric unit, suggesting that 19 C-terminal residues may have been proteolytically removed during crystallization. The fragment seen in the electron density maps (S1-E213) and the stable core fragment found to be resistant to proteolysis by Glu-C are identical. The V_M_ and the solvent content values calculated under the assumption that the C-terminus is absent rather than disordered are 2.1 Å^3^Da^−1^ and 45%, respectively, which falls within the range observed for protein crystals [27]. In contrast, the V_M_ value calculated under the assumption that there is one tetramer of full length YeaZ in the asymmetric unit, is 1.9 Å^3^Da^−1^, corresponding to a solvent content of approximately 36%. These values are at the low extreme of the range observed by Matthews for protein crystals. This confirmed that the crystals were formed by fragment 1–213 rather than full-length protein 1–232.

The model comprising residues 2–213 in each chain was built through iterative cycles of re-building with COOT and tight-NCS TLS refinement with Refmac [28], [29]. NCS and TLS groups corresponded to the four subunits in the asymmetric unit. Analysis of the stereochemical quality of the models was accomplished using MOLPROBITY [30]. Refinement statistics are given in Table 1.

**Table 1:** Refinement statistics.

Resolution range (Å)	79–3.1
Reflections	12,912
Residues/atoms	848/6,156
aR-factor	0.241
bFree R-factor	0.294
Average residual B after TLS refinement (Å^2^)	20.6
Bond-length deviation from ideality (Å)	0.015
Bond-angle deviation from ideality (°)	1.7
c ***Molprobity scores***	
Ramachandran space (%)	
Favored	84.0
Allowed	13.3
Outliers	2.7

a
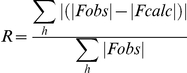
.

b The free R-factor was calculated on 5% of the data omitted at random.

c Reference [30].

### Accession codes

Coordinates and structure factors have been deposited to PDB RCSB with the accession code 3R6M.
